# Gene prediction of aging-related diseases based on DNN and Mashup

**DOI:** 10.1186/s12859-021-04518-5

**Published:** 2021-12-17

**Authors:** Junhua Ye, Shunfang Wang, Xin Yang, Xianjun Tang

**Affiliations:** School of Information Science and Engineering, Yunnan University, KunMing, 650000 China

**Keywords:** Gene prediction, Gene ontology, Mashup, Deep neural networks, Protein–protein interaction

## Abstract

**Background:**

At present, the bioinformatics research on the relationship between aging-related diseases and genes is mainly through the establishment of a machine learning multi-label model to classify each gene. Most of the existing methods for predicting pathogenic genes mainly rely on specific types of gene features, or directly encode multiple features with different dimensions, use the same encoder to concatenate and predict the final results, which will be subject to many limitations in the applicability of the algorithm. Possible shortcomings of the above include: incomplete coverage of gene features by a single type of biomics data, overfitting of small dimensional datasets by a single encoder, or underfitting of larger dimensional datasets.

**Methods:**

We use the known gene disease association data and gene descriptors, such as gene ontology terms (GO), protein interaction data (PPI), PathDIP, Kyoto Encyclopedia of genes and genomes Genes (KEGG), etc, as input for deep learning to predict the association between genes and diseases. Our innovation is to use Mashup algorithm to reduce the dimensionality of PPI, GO and other large biological networks, and add new pathway data in KEGG database, and then combine a variety of biological information sources through modular Deep Neural Network (DNN) to predict the genes related to aging diseases.

**Result and conclusion:**

The results show that our algorithm is more effective than the standard neural network algorithm (the Area Under the ROC curve from 0.8795 to 0.9153), gradient enhanced tree classifier and logistic regression classifier. In this paper, we firstly use DNN to learn the similar genes associated with the known diseases from the complex multi-dimensional feature space, and then provide the evidence that the assumed genes are associated with a certain disease.

**Supplementary Information:**

The online version contains supplementary material available at 10.1186/s12859-021-04518-5.

## Background

With the increase of aging population, countries all over the world invest more and more in the diseases of the elderly. This also caused more researchers to work to slow down the huge medical costs and social burden caused by human aging. Statistics show that the disease spectrum of human beings is changing from infectious diseases to chronic non infectious diseases such as hypertension, heart disease, stroke and cancer.

One of the main problems in the study of human aging is that it is much more difficult to carry out experiments due to obvious ethical reasons and long experimental time, so animal models with shorter life are usually preferred. This problem also creates the opportunity to deploy bioinformatics methods to study human aging. There is a large amount of data on this subject in the publicly available gene or protein database, but such data has not been fully developed [1, 2]. The purpose of this work is to analyze the human genes related to aging by combining various kinds of biological data and using deep learning data mining method. After we find the genes related to aging, we can use drugs to repair specific genes to delay human aging and treat human age-related diseases more effectively, which can alleviate the growing pressure of aging society [3]. Therefore, it is particularly important to propose a more effective multi label learning algorithm to reduce the workload of experimental verification in the field of biology [4, 5].

There are three contributions of this paper: (1) Using Mashup algorithm to reduce the dimensionality of biological network, and applying the processed data to the prediction of aging disease-related genes [6]; (2) Proposing a new DNN model; (3) According to the prediction of our algorithm, some negative genes close to known positive genes are recommended for further study for each disease. Our proposed architecture is called MDL (Mashup and Deep Learning), which integrates multiple data sources to obtain the prediction of the final model. We compare MDL with existing deep learning architecture [7], gradient boosting tree (GBT) [8]classifier (lightGBM Implementation) and traditional logistic regression (LR) classifier in terms of prediction ability [9].

The rest of this paper is organized as follows: Firstly, describes the construction principle of MDL and the method of compiling data. And then reports our experimental results, including the statistical analysis of the prediction performance of MDL, DNN, GBT and LR classifiers. The priority of disease genes is also the focus of our task, someone proposed Hetesim’s method in 2017 [10]. Especially, in the end of this part, a number of promising genes for further analysis are listed based on our method. Finally, in the discussion part, we use hypothesis testing to verify the advantages of the recommended gene compared with other genes. Then, according to the rules of statistical genes, the possibility of further prediction of aging related genes is prospected.

## Methods

### Overall structure

**Fig. 1:**
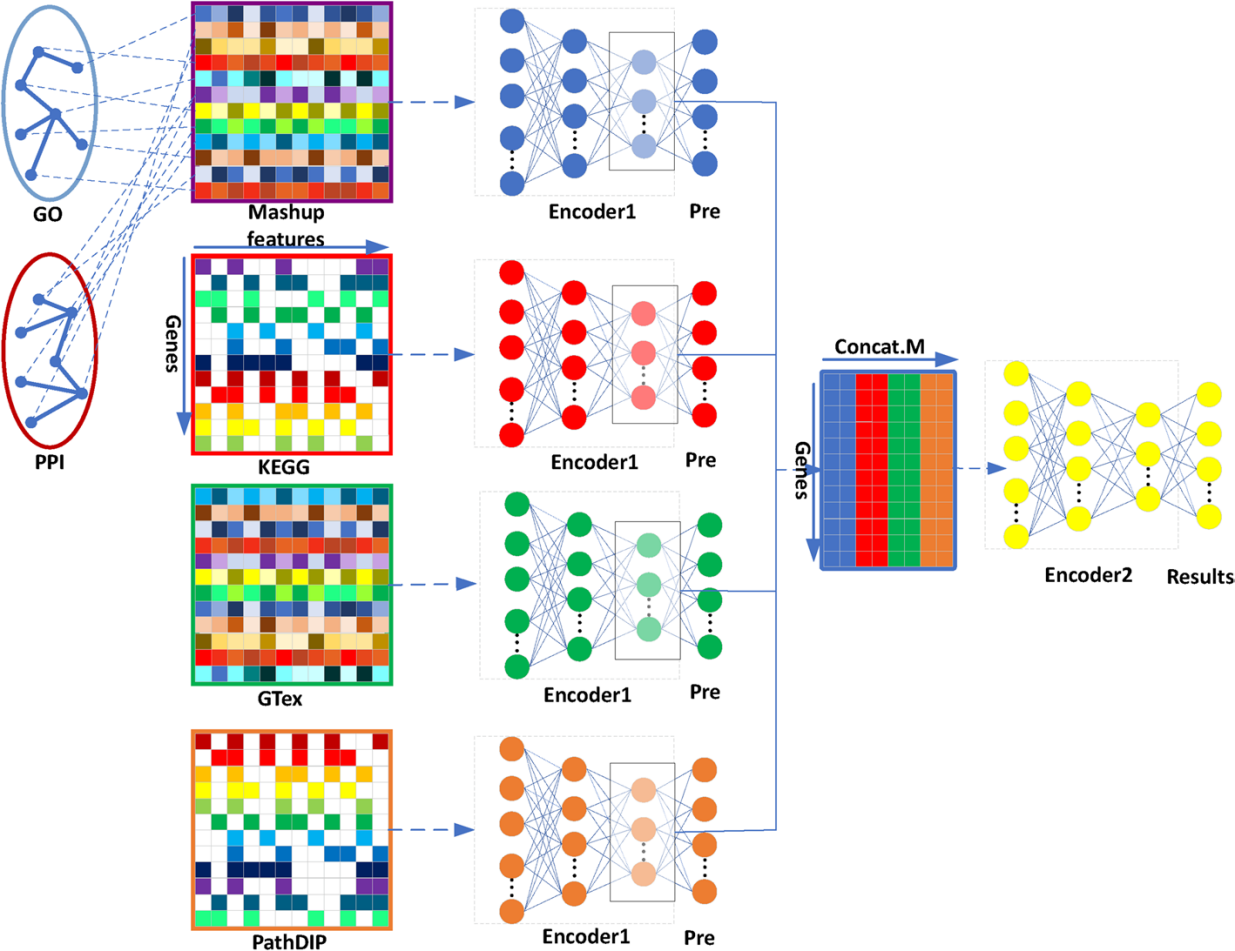
Algorithm frameworks. The vector of binary features consisted of 4 main parts (PPI, GO, KEGG, PathDIP), each part of which was equivalent to one of the data sources. The information for each data source was a boolean value, and if any gene contained this value, it scored 1, and otherwise, it scored 0. The remaining part (Mashup, GTEx) is non-BOOL value

In this paper, we use several types of features (or datasets), as shown in Fig. 1. The KEGG dataset, consisting of gene pathway information from the Kyoto Encyclopedia of Genes and Genomes, was used as described in Fabris and Freitas [11]. We obtained protein–protein interaction (PPI) features representing the human protein interaction network extracted from the STRING database v9.1 [12] and the Munich Information Center for Protein Sequences (MIPS). The Gene Ontology (GO) features were obtained by downloading data from the GO database [13]. Our method uses the Mashup algorithm [6] and the modular Deep Neural Network (DNN) algorithm proposed in Fabris et al. [7], as summarized in Fig. 1. Firstly, we used the Mashup algorithm to embed the GO terms and PPI features into an 800-dimensional feature vector, generating the Mashup dataset. Second, we use the modular DNN algorithm to learn a model in two phases. In the first phase we train 4 neural networks, denoted as “Encoder 1” in Fig. 1, each trained with a different type of feature (dataset)—namely the Mashup, KEGG, GTEx and PathDIP. Each Encoder 1 is used to reduce the dimensionality of its corresponding dataset. In the second phase, a neural network (denoted as “Encoder 2” in Fig. 1) is trained with a concatenation of the outputs of all trained Encoder 1 neural networks, generating a final predictive model. The specific model is as Fig. 1.

### Mashup

The high-dimensional nature of biological networks and the incompleteness of original networks bring great challenges to the adaptability of machine learning algorithms [14]. Specifically, first, the conflict between the huge amount of computation and the insufficient computation of most current computing devices. Second, the dimensions of multiple biological networks are not of the same order or magnitude, which makes it difficult to integrate multiple biological networks and predict gene function. Therefore, we need an algorithm to merge high-dimensional networks and reduce them into low dimensional data that is basically consistent with other smaller networks. On condition that, we can use an unified deep learning model to learn and predict multiple biological networks, and Mashup is such an excellent algorithm, which can well solve the above challenging problems efficiently.

Mashup algorithm is a network structure analysis tool based on random walk with restart (RWR). RWR is to start random walk again in each step of expansion with a certain probability, so as to identify the important nodes in the local and global topology of the network. The RWR from node i is defined as Eqs. (1, 2, 3):

1 $$\begin{aligned} s_{i}^{t+1}=\left( 1-p_{r}\right) B s_{i}^{t}+p_{r} e_{i}, \end{aligned}$$

2 $$\begin{aligned} B_{i j}=\frac{A_{i j}}{\sum _{i} A_{i j}}, \end{aligned}$$

3 $$\begin{aligned} e_{i}(j)=\left\{ \begin{array}{ll}1, &{} i=j \\ 0, &{} i \ne j\end{array}\right. \end{aligned}$$

where $s_i^t$ is a n-dimensional distribution vector, which holds the probability of reaching each node from *i* after t-step random walk; $p_{r}$ is the probability of restart; $e_i$ is a n-dimensional distribution vector. Each $B_{ij}$ of *B* represents the probability of a transition from node *i* to node *j*; *A* represents the n-dimensional weighted adjacency matrix of the original biological network containing *n* genes or proteins.

Due to the high dimensionality and incompleteness of biological network, there is a lot of noise in the node diffusion state obtained by restart random walk algorithm. In order to reduce the noise and dimensionality of data, each diffusion state is approximated by a polynomial logic model based on a lower dimensionality latent vector of nodes to generate a much smaller dimensionality than the original feature network.

When there are multiple networks, the above steps are firstly carried out for each network respectively, then the same weights used fuse each network into a final one. The final generation network is a 800 dimensional vector, which is 95% lower than the original 15,000 dimensional protein interaction network and gene ontology network. The calculation of data analysis is greatly reduced.

### Deep neural network model

With the continuous improvement of computer computing resources, deep learning has also shown its great development potential in various directions in bioinformatics [15, 16]. For example, Daniel et al. [17], Kulmanov et al. [18] used CNN and LSTM to predict nonprotein coding DNA sequences function, Skwark et al. [19] used deep learning network to predict protein structure, Gupta et al. [20] used deep architecture to learn the structure in gene expression data and applied it to gene clustering. It is believed that in the continuous development of deep learning, the prediction of gene expression can be further improved.

In general, the algorithm proposed in this paper has two stages, one is used to encode multiple biological data sources in the early stage, and to detect the coding effect, the other is used to train multiple biological data after serial coding, and give our prediction results. Because of the differences of data sources and data dimensionality, we propose two different deep learning models, Encoder1 and Encoder2.

In the model Encoder1, we use a four-layer fully connected neural network in each initial biological network, whose neurons in each layer are 64, 32, 32 and 16, respectively, and, the output is set to multi-label categories of 27 diseases. The Relu activation function is used in the four hidden layers to ensure that the output is a nonlinear combination of inputs, and add a bias neuron to improve the fitting ability of neurons. In the final output layer, we set tanh as the activation function. The combined training of Tanh and Relu activation functions can significantly improve the overall performance of the model [21]. In order to reduce the amount of parameters needed to be calculated in the training model, the learning rate is set to 0.001, and the neuron dropout rate of each layer to 0.5. Dropout can prevent over fitting by randomly sets input units to 0 with a frequency of rate at each step during training time. At the end of the training, we keep the last full connection layer data generated by each data set after model training, and then proceed to the next step. In this step of the model Encoder1, the prediction effect of each data set on the disease to be predicted can be initially seen. Although the prediction effect is not the final effect, it can help us measure the embedding effect of this model on each data set. The parameters needed to be trained for each data set are as follows: $Para1=n\times \left( 64+1\right) +64\times \left( 32+1\right) +32\times \left( 32+1\right) +32\times (16+1)+16\times 27$, where *n* is the dimensionality of each dataset.

In the final prediction model Encoder2, we use a deep neural network with three hidden layers. The neurons in each layer are 32, 24 and 16, respectively. Bias neurons are added to each layer. As mentioned above, the output is still a multi label vector of length 27. In the activation function, we refer to the data embedding model, using Relu in the hidden layer and tanh in the final output layer. The data we need to input is the data generated by each data set reserved in the previous model, which is connected in series, and then the model is trained. The training parameters of the model are as follows: $Para2=16\times 4\times \left( 32+1\right) +32\times (24+1)+24\times (16+1)+16\times 27=3{,}436{,}992$. Table 1 lists the hyperparameters of the deep learning model of Encoder1 and Encoder2.

**Table 1:** Learning hyperparameters for each network model

Models	Batch-size	Optimizer	Learning-rate	Epoch
Encoder1	2048	Adam	0.0001	150
Encoder2	3072	Adam	0.0001	150

## Results

### Comparison of MDL with other methods

In this section, we compare our traditional machine learning and deep learning algorithm with several other baseline algorithms, including: (1) existing DNN algorithm, including using a single feature type and concatenating all data sets for training; (2) gradient boosting tree algorithm; (3) logistic regression classifier (LR), using L2 regularization and other default parameters; (4) In order to verify the effectiveness of the Mashup algorithm, we compare the predictive performance of the DNN algorithm using an Encoder 1 module where Mashup was used to process the GO and PPI features together against the DNN algorithm without using Mashup (i.e. using a separate Encoder 1 module for each of the GO and PPI datasets). (5) Compared with the existing deep learning algorithm, the fitting degree of each module and the final prediction effect are compared. (6) The main idea of the Naive classifier is to think that the more research on a gene, the more likely it is to be related to disease. For the GO, PPI and PathDIP datasets, the degree of research is defined as the number of terms. If this gene does not exist in the data set, it is considered that its research level is 0. For data sets with non-BOOL values such as GTEx, the degree of genetic research that exists is set to 1, and the non-existent is set to 0. Finally, the research level of each gene in the 4 data sets is added together to generate a Naive classifier containing 4 types of feature information to identify whether our algorithm is simply assigning positive classification labels to genes with higher research levels.

Table 2 shows the information of each feature set. Unknown proportion refers to the proportion of the feature set that is not included in the label set. (e.g. proportion of genes without any PPI values, for the PPI feature.) Concat. M is the combination of PathDIP+GTEx+Mashup+KEGG, and Concat. P&G is the combination of GO + PPI + GTEx + KEGG + PathDIP. It should be noted that the Concat. P&G row of DNN column and naive column data in the Tables 3 and 4 does not contain KEGG and Mashup data.

**Table 2:** Data information of each feature set

Datasets	GO	PPI	PathDIP	GTEx	Mashup	KEGG	Concat. M	Concat. P&G
Unknown proportion	4%	19.4%	16.8%	4.1%	5.3%	3.7%	0	0
Data dimension	13,615	13,887	4790	84	800	318	5992	32,694
Sample size	18,418	15,469	15,956	18,597	15,709	7040	19,188	19,188

Finally, we use the area enclosed by the ROC curve under 10-folds cross validation. The predictive accuracy measure (AUC value in this case) is calculated as the average of the measure in the test set of 10 experiments. Therefore, each data instance is used once in the test set and nine times in the training set. AUC is the area under the ROC curve and surrounded by the coordinate axis, while the abscissa of the ROC curve is the false positive rate (FPR), and the ordinate is the true positive rate (TPR). Their calculation formulas are as Eq. (4):

4 $$\begin{aligned}&{\mathrm {FPR}}=\frac{F P}{F P+T N}, \mathrm {TPR}=\frac{T P}{T P+F N} . \end{aligned}$$

where FN, FP, TN, TP mean false negative, false positive, true negative and true positive, respectively. Because some genes have unknown values of features for each type of feature, we use two different strategies to generate the training and test sets according to whether or not genes with unknown features are all included in those datasets. Tables 3 and 4 show the value of the AUROC measure obtained by each method, for the datasets containing or not (respectively) the genes with unknown values of the corresponding type of feature. The bold in tables 3 and 4 represents the prediction results of the best algorithm in the corresponding dataset.

**Table 3:** AUROC values obtained by 10-fold cross-validation in datasets with unknown genes

Algorithms/datasets	Encoder1	GBT	LR	DNN	Naive
PPI	0.7451	0.6585	0.6844	0.6381	**0.7995**
GO	0.8326	0.8520	0.7900	**0.8498**	0.6080
GTEx	**0.8165**	0.7156	0.7173	0.7535	0.5062
pathDIP	**0.8457**	0.8392	0.7600	0.7507	0.7817
Mashup	**0.8637**	NA	NA	NA	NA
KEGG	**0.8322**	NA	NA	NA	NA

**Table 4:** AUROC values obtained by 10-fold cross-validation in datasets without unknown genes

Algorithms/datasets	Encoder1	GBT	LR	DNN	Encoder2
PPI	**0.7464**	0.6782	0.6889	0.6897	NA
GO	0.8321	0.8268	0.7981	**0.8583**	NA
GTEx	**0.8163**	0.7114	0.7233	0.7476	NA
pathDIP	**0.8420**	0.8314	0.7607	0.8051	NA
Mashup	**0.8631**	NA	NA	NA	NA
KEGG	**0.8324**	NA	NA	NA	NA
Concat. M	0.8881	0.8688	0.8760	NA	**0.9153**
Concat. P&G	0.7995	0.8503	0.8777	**0.8795**	**0.9095**

It should be noted that the data in the first four rows (PPI, GO, GTEx, PathDIP) of columns 3–5 (GBT, LR, DNN) in Tables 3 and 4 are provided by Fabris et al. [7]. From the data in the above two tables, it can be seen that the effect of only containing known genes is better than that of containing unknown genes. In Table 3, when using only the PPI data set, our Encoder1 is 5.44% lower than Navie, but it is more than 6% higher than other algorithms, and more than 10% higher than the previous DNN algorithm. When using the GO data set alone, Encoder1 is slightly lower than DNN. When using other data sets, our algorithm will perform better than other algorithms.

In Table 4, we use Mashup algorithm combined with the MDL model, which has more advantages than the existing deep learning algorithm or the traditional machine learning algorithms such as logistic regression algorithm and gradient boosting tree. When we directly use PPI or GO data to input Encoder1, too large data dimensionality leads to a huge increase in the amount of calculation, and the convergence speed is also very slow (10-fold cross-validation training time is about 10 h per module). When we use the Mashup algorithm to combine PPI and GO, it not only greatly reduces the amount of machine learning computation, but also improves the prediction effect. From 0.7464 of PPI and 0.8321 of GO to 0.8631 of Mashup. Then we add all the data sets into Encoder2, that is, we bring them into Concat. P&G respectively to make the final prediction. Although our data sample dimensions are larger (16 * 5 vs 16 * 4, where 16 is the each feature data dimensions after Dimensionality reduction by Encoder1), we still have advantages in the prediction effect (0.9095 vs 0.8795). It proves that our deep learning model has better effect than the previous deep learning model. Finally, by comparing the training effect of encoder2 using concat. M and concat. P&G data sets, we find that the use of concat. M also has a significant improvement (0.9153 vs 0.9095). This shows that mashup algorithm not only has a good effect in embedding PPI and GO, but also has a positive effect in integrating it into other feature data sets.

In order to see the advantages of our method more intuitively, We draw ROCs in each method in the case of using Concat. M or Concat. P&G datasets. It is worth noting that the larger the area under the curve, that is, the higher the curve is, the better the prediction effect of the corresponding algorithm.

**Fig. 2:**
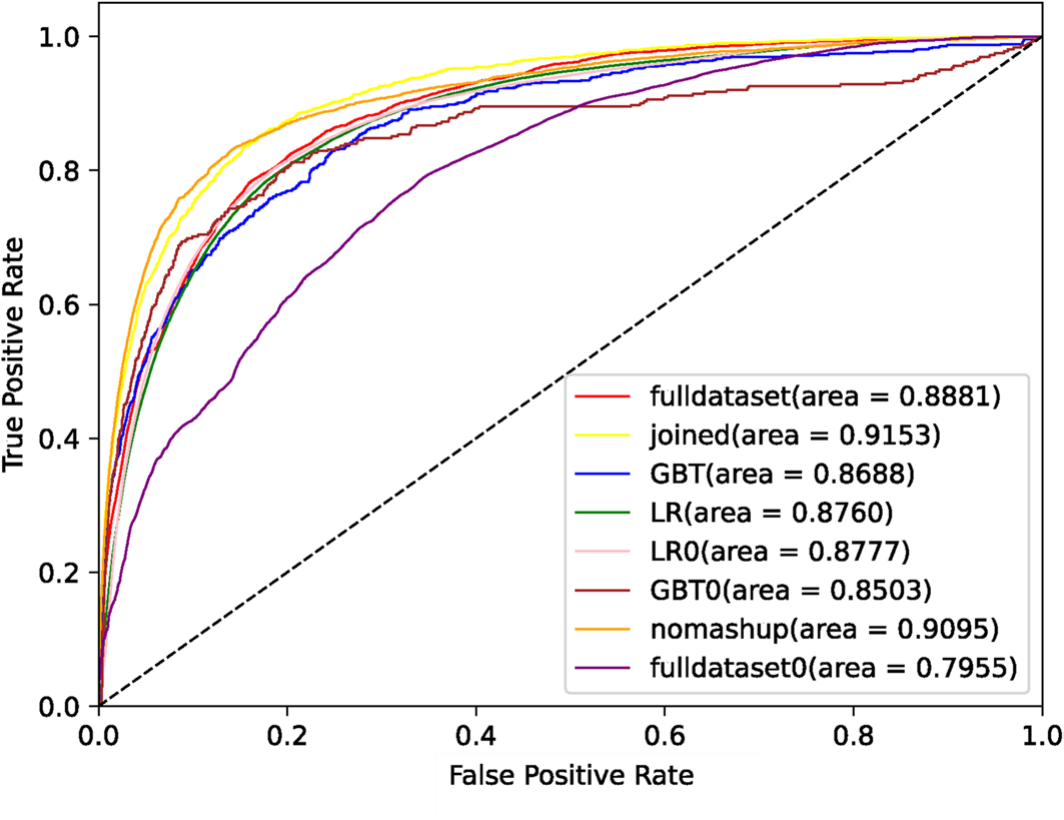
Comparison of ROCs by Concat. M datasets and Concat. P&G datasets on each methods

The corresponding results of Fig. 2 are the rows concat. M and concat. P&G in Table 4. GBT, GBT0 use GBT algorithm, LR, LR0 use LR algorithm, fulldataset and fulldataset0 only use Encoder1 algorithm, the data set Concat. M is used if the suffix of their names contains 0, otherwise Concat. P&G is used. Joined and nomashup correspond to the two results of whether our MDL algorithm uses Mashup for embedding in the dataset PPI and GO. In Fig. 2, we can see that ‘joined’ is significantly higher than all other methods except ‘nomashup’. We can also see that directly concatenating several large data sets together using Encoder1 will also seriously reduce the training effect.

### Results statistical analysis

We use our own method to compare existing DNN algorithms, GBT and logistic regression classification, and make a statistical analysis in the case of considering the use of known and unknown genes. Considering that the data set used in a single module is different from that used in the combination of all our data, and the prediction performance is obviously better in the case of combining multiple data sets, we do not give statistical analysis of the results for the individual datasets (types of features), and focus instead on the statistical analysis for the results combining all datasets. Our statistical analysis method includes two kinds, one is based on T-test, which is used to test whether there is significant difference in the mean value of cross validation results. We add the statistical significance test based on Mann–Whitney-U test to supplement. In the U-test, we calculate the *p* value of “Greater”, “Two side” and “Less” models, that is, the null hypothesis is that other methods are better, the same or worse than ours. The data we tested and analyzed were 10-folds cross validation of 30 random seed changes, and the AUC value predicted by each fold record was verified.

The data in Table 5 shows the comparison results between our method and other methods. The first column lists our method and each method that needs to be compared. The second column is the combination method of each characteristic data set under different methods. The third column is the corresponding average AUROC value. The fourth column gives the significance test results of T hypothesis. The fifth column is the statistical results based on Mann–Whitney-U test. From this table, we can see that except for the prediction results in the line of Encoder2, other methods can easily reject the null hypothesis through *p* value and get the conclusion that our method works better. However, using Encoder2 to combine data with Concat. P&G method can not reject the null hypothesis of T-test ($0.1256<0.05$), and their average value has no significant difference. At the same time, in Mann–Whitney-U test, the p value of Encoder2 algorithm in Concat. P&G is better than Concat. M is 0.0261, which can reject the null hypothesis. Therefore, it can be said that at least we embed PPI and GO datasets in the way of Mashup, and then use our deep learning algorithm will not be worse than using PPI and GO datasets directly.

**Table 5:** Comparing the MDL approach (AUROC = 0.9153) with each data set of others methods using T-test and Mann–Whitney-U hypothesis testing

Algorithms	Feature combination	AUROC	T-test	Mann–Whitney-U test ***p*** value		
			*p* value	Greater	Two-side	Less
Encoder1	Concat. M	0.8881	0.0045	0.0021	0.0043	0.9980
	Concat. P&G	0.7955	1.721e−09	4.428e−08	8.857e−08	1.0000
Encoder2	Concat. P&G	0.9095	0.1256	0.0261	0.0522	0.9739
GBT	Concat. M	0.7301	6.778e−09	4.523e−08	9.046e−08	1.0000
	Concat. P&G	0.8503	0.0001	4.146e−05	8.292e−05	1.0000
LR	Concat. M	0.8760	1.704e−06	1.461e−07	2.922e−07	1.0000
	Concat. P&G	0.8777	4.071e−07	1.094e−07	2.189e−07	1.0000

### AUROC results of individual diseases

**Fig. 3:**
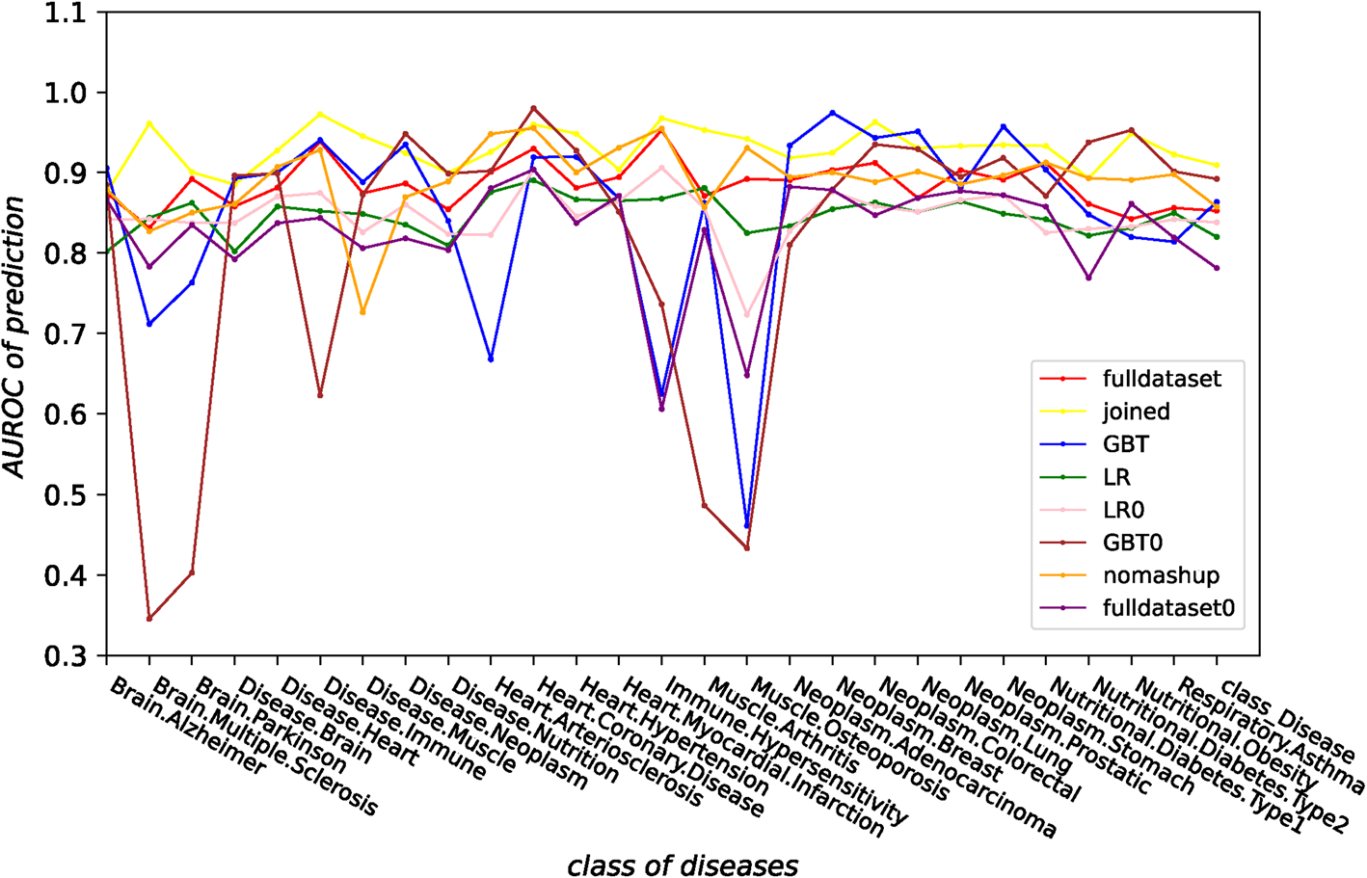
AUROC value of 27 diseases predicted by each algorithm

In the end, we calculate by prediction AUROC of each class label (disease) for the final results predicted by all methods, and the AUROC is shown in Fig. 3. As we can see from Fig. 3 that the stability of prediction AUROC for each disease is the best among all methods, and our algorithm has the best performance in most diseases.

### Analysis of prediction results

In the last step of prediction, we rank each disease score corrsponding to each gene in order to identify the positive (with disease) class labels recommended by the model. The set of samples (genes) predicted as having a positive class label by the model are likely to contain some negative samples (i.e. the corresponding diseases are not annotated), and these negative samples are the objects that we need to further study [22, 23]. Because the genes without annotation information are regarded as negative labeled samples by default, the originally positive samples may be wrongly annotated as negative in the dataset. It seems that the reliability of negative label is low, and the lack of relevant evidence does not mean that there is no evidence of disease [24].

In order to identify these negative genes more reliably, we carried out the following rule screening. We ranked all the negative genes according to the predicted probability that the gene is associated with each disease (i.e. the predicted probability of each positive class label), and selected the top 30 genes with negative labels with the highest probability, since the disease (class label) with less annotated genes, osteoporosis, contains only 30 genes, then we chose to recommend 30 genes for each disease. Since there is generally around 15,000 genes in the sample, which means at least the top 0.2%, i.e. 30/15,000, of the predicted genes met the requirements. Because of the randomness of deep learning, we repeated this experiment for 30 times, and required at least 20 times to meet the above requirements before we could get our recommendation for negative label samples. In fact, all the 30 recommended genes met this preset standards, furthermore, among the top 30 genes there are at least 25 genes were listed in the practical process of 30 repeats, exceeding the above standards.

Due to the limitation of space, instead of listing all the 30 positive genes, We only listed the top five genes in three of the 27 diseases. The 27 kinds of labels (diseases) include one primary label(Class_Disease), seven secondary labels(Disease.Brain, Disease.Heart, Disease.Immune, Disease.Muscle, Disease.Neoplasm, Disease.Nutrition, Disease.Respiratory.Asthma), and the remaining 19 are tertiary labels. The secondary label is defined as: if a gene associated with any one of the tertiary labels (disease) of the corresponding class, the secondary label is associated with the gene. Primary label is the same. We have chosen three of the seven more comprehensive secondary labels (Disease.Immune, Disease.Brain, Disease.Nutritional). Giving their average probability (Ave. P), the lowest probability (Low. P) and the highest probability (Hig. P) of positive prediction according to the frequency of occurrence in 30 repeated experiments. These results are summarized in Table 6, in which column 1 is the class of disease, column 2 is the Entrez ID of the gene and the official abbreviation of the name and the official full name. column 3–5 is the average, highest, lowest probability of recommended gene in 30 experiments. Column 6 is the average probability of all negative genes except as recommended (top 30) genes. As can be seen from Table 6, the probability that the genes we selected will be positive according to the algorithm is far greater than other genes.

**Table 6:** The top five genes related to each of the three diseases according to the frequency of occurrence in 30 repeated experiments, and the various probabilities of association with disease of these candidate genes

Disease	Candidate genes	Ave. P	Hig. P	Low. P	Ave. P of rest genes
Immune	3553(*IL1B*—interleukin 1 beta)	0.119864	0.166426	0.062936	0.00128
	1544(*CYP1A2*—cytochrome P450	0.115728	0.159259	0.061422	
	family 1 subfamily A member 2)				
	4023(*LPL*— lipoprotein lipase)	0.113289	0.155988	0.06022	
	1543(*CYP1A1*— cytochrome P450	0.111613	0.154343	0.059018	
	family 1 subfamily A member 1)				
	4846(*NOS3*—nitric oxide synthase 3)	0.110097	0.154304	0.057448	
Brain	1544(*CYP1A2*—cytochrome P450	0.204762	0.303633	0.111395	0.0363
	family 1 subfamily A member 2)				
	1559(*CYP2C9*—cytochrome P450	0.190703	0.28241	0.104468	
	family 2 subfamily C member 9)				
	1586(*CYP17A1*—cytochrome P450	0.175779	0.243321	0.104167	
	family 17 subfamily A member 1)				
	338(*APOB*—apolipoprotein B)	0.174123	0.256506	0.097296	
	1557(*CYP2C19*—cytochrome P450	0.173544	0.258927	0.0953	
	family 2 subfamily C member 19)				
Nutrition	3553(*IL1B*—interleukin 1 beta)	0.343483	0.496965	0.195028	0.00677
	1544(*CYP1A2*—cytochrome P450	0.332656	0.478875	0.190983	
	family 1 subfamily A member 2)				
	1557(*CYP2C19*—cytochrome P450	0.284008	0.414314	0.163521	
	family 2 subfamily C member 19)				
	4035(*LRP1*—LDL receptor)	0.280427	0.412398	0.159798	
	related protein 1				
	2688(*GH1*—growth hormone 1)	0.279018	0.381114	0.171254	

Considering that the significance of comparing the best 30 genes recommended by the algorithm with the average value of all other negative genes may not be comprehensive enough (other genes may contain a lot of samples with low recommendation values, thus pulling down some samples similar to the recommended genes). So we used the same method of selecting recommended genes to select genes with probabilities ranging from 31 to 60 for each disease. And then we use the probability of their association with the disease and the top 30 genes for hypothesis testing. The null hypothesis is that the gene we recommend is the same as his neighbor.

It can be seen from Table 7 that the probability of our top 30 genes is significantly better than that of our neighbor genes (ranking 31–60). It should be noted that the statistics can’t show that the negative class-label genes ranked after the 30th have no relationship with the diseases we study. On the contrary, if the genes ranked after the 30th have evidence related to a certain disease, according to our algorithm, the top 30 genes recommended by us should also have a higher probability of obtaining evidence related to diseases.

**Table 7:** Testing results for the statistical hypothesis of whether the recommended 30 genes are significantly different from the subsequent 30 genes for 27 aging-related diseases

Disease classification	*p* values	Disease classification	***p*** values
Disease.Brain	8.484e−09	Disease.Neoplasm	2.033e−09
Brain.Alzheimer	3.474e−10	Neoplasm.Adenocarcinoma	1.464e−10
Brain.Multiple.Sclerosis	5.967e−09	Neoplasm.Breast	1.205e−10
Brain.Parkinson	1.070e−09	Neoplasm.Colorectal	2.154e−10
Disease.Heart	5.072e−09	Neoplasm.Lung	7.380e−10
Heart.Arteriosclerosis	1.776e−10	Neoplasm.Prostatic	3.196e−09
Heart.Coronary.Disease	6.695e−11	Neoplasm.Stomach	2.609e−10
Heart.Hypertension	4.504e−11	Disease.Nutrition	7.118e−09
Heart.Coronary.Disease	6.695e−11	Nutritional.Diabetes.Type1	3.689e−11
Disease.Immune	6.065e−10	Nutritional.Diabetes.Type2	2.227e−09
Immune.Hypersensitivity	5.494e−11	Nutritional.Obesity	4.975e−11
Disease.Muscle	2.438e−11	Disease.Respiratory.Asthma	7.380e−10
Muscle.Arthritis	1.464e−10	Class_Disease	2.831e−08
Muscle.Osteoporosis	8.484e−09		

## Discussion

In fact, the evidence of genes and related diseases that we recommend can also be found in a large number of literatures. For example, *IL1B* of immune related diseases was approved by Zhi-gang et al. [25]. Found that it is related to immune T cells in abortion of pregnant women, and found in the paper 2016 that the lineal homologous gene of *IL1B* also plays an important role in the immune regulation of fish [26]. Liver *IL1B* may be a new target for restoring bile and sterol transport to treat PNAC [27, 28]. The specific expression of *CYP2C9* in the axons and synapses of brain neurons may be related to some brain diseases, and the mutation allele frequency of *CYP2C9* is higher in 23 tumor samples, such as breast tumor and brain tumor, which indicates that *CYP2C9* may be related to tumor [29, 30].

The evidence we found is not limited to these genes. With the help of Google academic, we can easily find the research papers related to the recommended genes for each disease. For the 15 genes mentioned above, we list the relevant information in the Table 8. Finally, only the evidence of *CYP2C19* gene recommended for nutritional diseases was not found.

**Table 8:** Evidence of 15 genes recommended for association with disease

Gene ID	Genes associated with “Disease.Immune”	Relevant evidence
3553	*IL1B*—interleukin 1 beta	Roghieh Safari et al. [26]
1544	*CYP1A2*—cytochrome P450 family 1	Klein et al. [31]
	subfamily A member	
4023	*LPL*—lipoprotein lipase	*S. aureus* et al. 2015 [32]
1543	*CYP1A1*—cytochrome P450 family 1	Uno et al. [33]
	subfamily A member 1	
4846	*NOS3*—nitric oxide synthase 3	Bogdan et al. [34]
Gene ID	Genes associated with “Disease.Brain ”	Relevant evidence
1544	*CYP1A2*—cytochrome P450 family 1	Siokas et al. [35]
	subfamily A member 2	
1559	*CYP2C9*—cytochrome P450 family 2	Sun et al. [36]
	subfamily C member 9	
1586	*CYP17A1*—cytochrome P450 family 17	Emanuelsson et al. [37]
	subfamily A member 1	
338	*APOB*—apolipoprotein B	Bjelik et al. [38]
1557	*CYP2C19*—cytochrome P450 family 2	Ingelman-Sundberg et al. [39]
	subfamily C member 19	
Gene ID	Genes associated with “Disease.Nutrition”	Relevant evidence
3553	*IL1B*—interleukin 1 beta	Norde et al. [40]
1544	*CYP1A2*—cytochrome P450 family 1	Agúndez et al. [41]
	subfamily A member 2	
1557	*CYP2C19*—cytochrome P450 family 2	None
	subfamily C member 19	
4035	*LRP1*—LDL receptor related protein 1	Masson et al. [42]
2688	*GH1*—growth hormone 1	Thissen et al. [43]

**Fig. 4:**
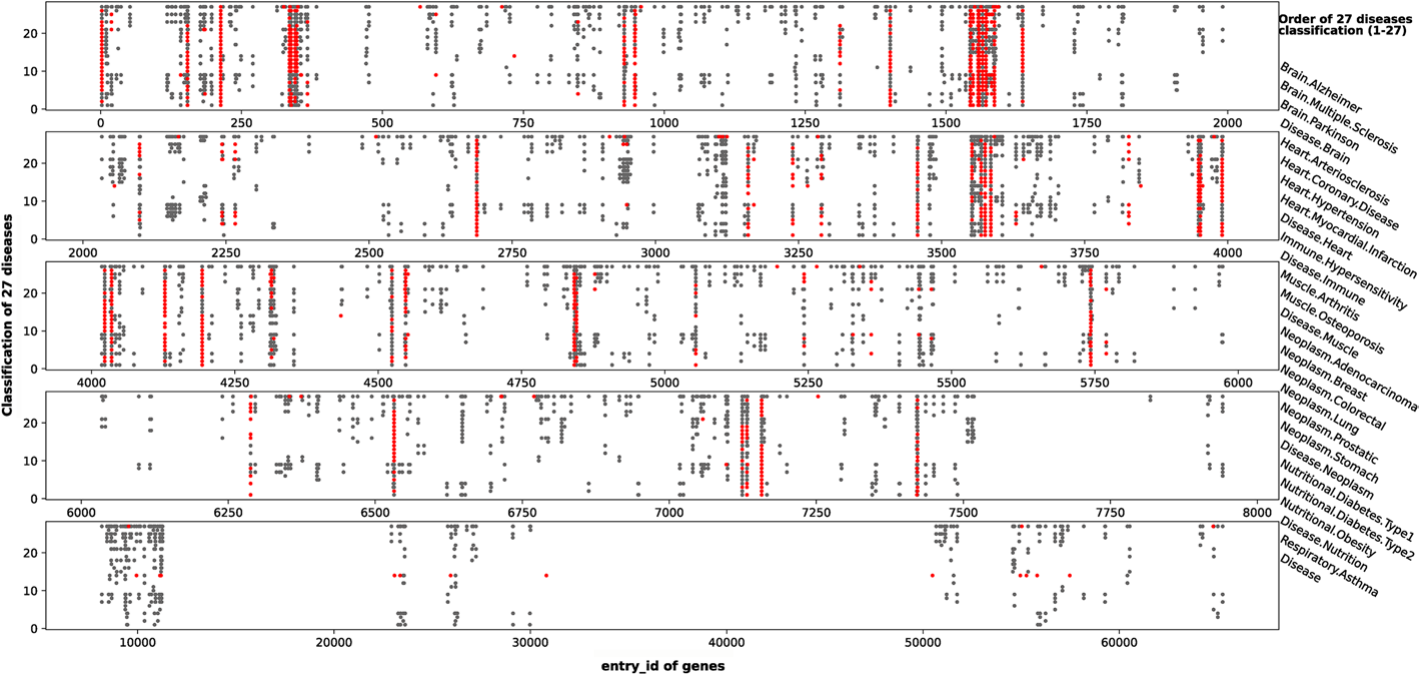
Distribution of recommended genes in 27 diseases. The gray dots in the figure represent the association between diseases and genes in the tag set, and the red dots represent the association between diseases and genes we predict

We have recommended 30 genes that are most likely to be positive for each disease. However, due to the limitation of space, we have only made a detailed analysis of five genes of three diseases (Disease.Immune, Disease.Brain, Disease.Nutritional). But we want to find out whether there is some internal relationship between these genes recommended for each disease, so as to find out the genes that may be related to aging. Therefore, we have made the Fig. 4 for all the recommended genes, so we can see their distribution more intuitively. Figure 4 should be viewed horizontally.

The Vertical-axis of the five subgraphs in Fig. 4 corresponds to 27 diseases. The order of the Vertical-axis from 1 to 27 corresponds to the order from top to bottom listed on the right of the figure. The Horizontal-axis of each subgraph corresponds to the ID value of the recommended gene.

As can be seen from the Fig. 4, the majority of the recommended genes are distributed within 1–8000, and after 8000 only the category of diseases exists with recommended genes. The distribution of these recommended genes in 1–8000 shows that many of our recommended genes like to cluster in multiple diseases. Although we have not made further analysis on each type of disease, it can be seen from the gene prediction results that it is more likely that similar diseases will predict the same associated genes. This phenomenon shows that the diseases we choose have certain internal relations, and as far as we know, the only thing they have in common may be that they are all related to aging. This also shows that our research can not only stay in the level related to aging diseases, but also have a certain reference value in the deeper level of the relationship between genes and aging. Some recommended genes appear frequently in some regions, such as 300–400, 1500–1600, 3500–3600, 7100–7200, and so on. It may be more helpful to reveal the relationship between genes and aging by giving priority to gene research in these regions. An additional file shows all recommended genes in more detail (see Additional file 1).

## Conclusion

In general, our method has a significant improvement in prediction performance compared with the existing modular deep learning model or the traditional machine learning method like LR or GBT in both single module prediction and total module prediction accuracy. At the same time, for a single module, we use Mashup algorithm to embed PPI and GO into a network, which greatly improves the original performance of only two modules. In addition, due to the decline of data dimensionality, the training time also has a great decline (from dozens of hours to only more than 1 h). We used 30 times of random seed replacement to repeat the experiment. The 30 negative marker genes were recommended for further analysis by biological researchers with more than 99.8% (i.e. $1-(30/19{,}188)$) positive probability. The hypothesis test of the top 30 genes in our experiment and the genes ranking 31–60 confirmed that the recommended genes are significantly different from other genes. We also found evidence from the literature that supports our assumption that genes may be disease-related, which further confirms that lack of evidence of gene-disease association does not mean they are not disease related.

## Supplementary Information

**Additional file 1:** Details of predicted genes.

## Data Availability

The source code and datasets located https://github.com/jhuaye/GenesRelateDiseases.

## Abbreviations

MDL: Mashup and deep learning
PPI: Protein–protein interactions
GO: Gene ontology
KEGG: Kyoto Encyclopedia of genes and genomes Genes
GTEx: The genotype-tissue expression (GTEx) project
